# Ocean acidification stimulates particulate organic carbon accumulation in two Antarctic diatom species under moderate and high natural solar radiation

**DOI:** 10.1111/jpy.12753

**Published:** 2018-06-25

**Authors:** Jasmin P. Heiden, Silke Thoms, Kai Bischof, Scarlett Trimborn

**Affiliations:** ^1^ Alfred Wegener Institute Helmholtz Center for Polar and Marine Research Am Handelshafen 12 27570 Bremerhaven Germany; ^2^ Marine Botany University Bremen Leobener Str. NW2 28359 Bremen Germany; ^3^ Alfred Wegener Institute Helmholtz Center for Polar and Marine Research Am Handelshafen 12 27568 Bremerhaven Germany

**Keywords:** climate change, CO_2_, light, multiple stressors, photophysiology, photosensitivity, phytoplankton, Southern Ocean

## Abstract

Impacts of rising atmospheric CO_2_ concentrations and increased daily irradiances from enhanced surface water stratification on phytoplankton physiology in the coastal Southern Ocean remain still unclear. Therefore, in the two Antarctic diatoms *Fragilariopsis curta* and *Odontella weissflogii*, the effects of moderate and high natural solar radiation combined with either ambient or future pCO_2_ on cellular particulate organic carbon (POC) contents and photophysiology were investigated. Results showed that increasing CO_2_ concentrations had greater impacts on diatom physiology than exposure to increasing solar radiation. Irrespective of the applied solar radiation regime, cellular POC quotas increased with future pCO_2_ in both diatoms. Lowered maximum quantum yields of photochemistry in PSII (F_v_/F_m_) indicated a higher photosensitivity under these conditions, being counteracted by increased cellular concentrations of functional photosynthetic reaction centers. Overall, our results suggest that both bloom‐forming Antarctic coastal diatoms might increase carbon contents under future pCO_2_ conditions despite reduced physiological fitness. This indicates a higher potential for primary productivity by the two diatom species with important implications for the CO_2_ sequestration potential of diatom communities in the future coastal Southern Ocean.

**Abbreviations**
[RCII]^cell^: concentration of functional photosystem II reaction centers per cell
absETR: absolute electron transport rate
C:N: carbon to nitrogen
CCM: carbon concentrating mechanism
e^−^: electron
ETR_m_: maximum absolute electron transport rate
F_q_’/F_m_’: effective photosystem II quantum yield under ambient light
F_v_/F_m_: dark‐adapted maximum photosystem II quantum yield
I_K_: minimum saturating irradiance
LHP: light‐harvesting pigments
LPP: light‐protective pigments
OA: ocean acidification
pCO_2_: carbon dioxide partial pressure
*p*: dark‐adapted connectivity factor of adjacent photosystem IIs
POC: particulate organic carbon
PON: particulate organic nitrogen
SO: Southern Ocean
α: maximum light‐use efficiency
σ_PSII_: dark‐adapted functional absorption cross‐section of photosystem II
τ_QA_: dark‐adapted re‐oxidation time of the electron acceptor Q_A_

The effects of increasing atmospheric carbon dioxide (CO_2_) concentrations from anthropogenic emissions and the subsequent increased CO_2_ uptake by the world's oceans are expected to have greater effects on polar oceans due to the higher solubility of CO_2_ at low temperatures (IPCC 2014). As a consequence, true aqueous CO_2_ concentrations in the future ocean will double by 2100 (IPCC 2014) potentially stimulating primary production particularly of cold waters such as the Southern Ocean (SO; Zeebe and Wolf‐Gladrow 2001, Orr et al. 2005). In today's ocean, photosynthetic carbon fixation by marine phytoplankton is constrained by low CO_2_ concentrations in ocean surface waters (Mackey et al. 2012). This is because diffusive uptake of CO_2_ by phytoplankton is insufficient to saturate their CO_2_ fixing enzyme ribulose‐1,5‐bisphosphate‐carboxylase‐oxygenase (RubisCO). To circumvent this, many phytoplankton species, including Antarctic diatoms (Trimborn et al. 2013), operate energy consuming carbon concentrating mechanisms (CCMs, Mackey et al. 2012). Thus, a reduced energy demand due to a down‐regulation of the CCM under high pCO_2_ can stimulate growth and carbon fixation of temperate phytoplankton species (e.g., Sobrino et al. 2008, McCarthy et al. 2012, Rokitta and Rost 2012, Li and Campbell 2013, Li et al. 2014). Under cold temperatures, however, such energy savings were not found to give much benefit to Antarctic phytoplankton (Kranz et al. 2015) as they require very high RubisCO concentrations in order to maintain maximum photosynthesis rates (Young et al. 2014). In line with this, there was no stimulating effect of high pCO_2_ on the growth of Antarctic phytoplankton species observed (Boelen et al. 2011, Hoogstraten et al. 2012, Trimborn et al. 2013, 2017, Hoppe et al. 2015, Heiden et al. 2016). In the two diatoms, *Fragilariopsis curta* and *Odontella weissflogii*, OA was found to decrease growth and particulate organic carbon (POC) production under low and medium, but not under high light (Heiden et al. 2016).

By the year 2100, next to rising atmospheric CO_2_ concentrations, sea surface temperatures are expected to increase thus enhancing sea‐ice melt and reducing seasonal ice cover by 14% (Russell et al. 2006, IPCC 2014). Warmer temperatures and increased freshwater input from sea‐ice melt might increase vertical stratification, which would reduce the mixed layer depth and increase daily mean irradiances (Bopp et al. 2001, Sarmiento et al. 2004, Boyd et al. 2015). Especially in coastal environments such as the Western Antarctic Peninsula (WAP), light conditions are very variable with phytoplankton communities often dominated by diatoms (Sarthou et al. 2005, Annett et al. 2010). The amount of light available to phytoplankton strongly affects growth and the rate of carbon fixation (Falkowski and Raven 2007). With increasing growth irradiances, Antarctic phytoplankton species have been shown to increase growth and carbon fixation, but only until photosynthesis is saturated (Fiala and Oriol 1990, Heiden et al. 2016). Under high irradiances, photosynthesis might get over‐saturated. To prevent photodamage under these conditions, phytoplankton cells usually dissipate excess energy via non‐photochemical pathways, including the rapidly acting xanthophyll cycle (Falkowski and Raven 2007, Brunet and Lavaud 2010, Goss and Jakob 2010). Electrons generated by photosystem II (PSII) can be transported linearly fueling carbon fixation by RubisCO in the Calvin–Benson cycle (Falkowski and Raven 2007). However, if the Calvin–Benson cycle is saturated electrons might get shed through alternative electron pathways such as the Mehler reaction, midstream oxidase pathways (Behrenfeld and Milligan 2012), and cyclic electron transport around photosystem I (PSI; Falk and Palmqvist 1992). Cyclic electron flow around PSI was recently found to be very active in Antarctic diatom‐dominated phytoplankton communities as well as in the sea‐ice diatom *Fragilariopsis cylindrus* (Goldman et al. 2015).

Under fluctuating light conditions, phytoplankton cells have to permanently adjust their photosynthetic apparatus to the changing conditions in order to optimize light capture and electron transfer and to prevent photodamage. Consequently, cellular energy demands increase, which can lower biomass build‐up (Wagner et al. 2006, 36 and 165 μmol photons · m^−2^ · s^−1^; Shatwell et al. 2012, 10–130 and 200–1,300 μmol photons · m^−2^ · s^−1^; Su et al. 2012, 40–50 μmol photons · m^−2^ · s^−1^; Hoppe et al. 2015, 90 μmol photons · m^−2^ · s^−1^; Lepetit et al. 2016, 40, 80 and 158 μmol photons · m^−2^ · s^−1^). Under dynamic compared to sine light of the same daily integrated irradiance, growth and cellular POC quotas remained unaltered in the Antarctic diatom *Chaetoceros brevis* (Boelen et al. 2011, 76 μmol photons · m^−2^ · s^−1^), but were lowered under constant compared to dynamic light in *Chaetoceros debilis* (Hoppe et al. 2015, 90 μmol photons · m^−2^ · s^−1^).

With increasing daily mean fluctuating irradiances, growth and POC quotas were enhanced in *Chaetoceros brevis* (Boelen et al. 2011, 76 and 200 μmol photons · m^−2^ · s^−1^) while they remained unaffected for the Antarctic diatom *Fragilariopsis cylindrus* (Mills et al. 2010, 125 and 250 μmol photons · m^−2^ · s^−1^). The combination of increasing dynamic light with elevated pCO_2_ led to a stimulation of growth under moderate, but not under high dynamic light in *C. brevis* (Boelen et al. 2011, 76 and 200 μmol photons · m^−2^ · s^−1^). In comparison, negative effects of OA on growth and POC production were already found under the moderate dynamic irradiance of 90 μmol photons · m^−2^ · s^−1^ in *C. debilis* (Hoppe et al. 2015). Thus, responses of Antarctic diatoms to future pCO_2_ were found to differ between constant and dynamic light and might further depend on the intensity of the applied daily mean irradiance.

In a preceding study, growth and POC production of * Fragilariopsis curta* were highest under low light, indicating a saturation of photosynthesis while for *Odontella weissflogii* highest growth and POC production rates were reached only at medium irradiance (Heiden et al. 2016, low = 20, medium = 200 and high = 500 μmol photons · m^−2^ · s^−1^). In this study, we aimed to investigate whether the observed physiological responses to increasing constant irradiances are also representative for natural light regimes. Therefore, the two species were grown outdoors under natural fluctuating solar irradiances of two different intensities, exposing them to more realistic light conditions. In response to OA, growth and POC production rates were previously reported to decrease in these species under low and medium, but not high constant light (Heiden et al. 2016). In response to OA and high irradiance, they further exhibited an increased photosensitivity (Heiden et al. 2016), which was also found for other Antarctic diatom species under these conditions (Trimborn et al. 2017). To deepen our understanding of OA effects in response to increasing dynamic light, *Fragilariopsis curta* and *Odontella weissflogii* were exposed to current and future pCO_2_ in combination with moderate and high solar radiation (HSR), applying more realistic climate change scenarios. The physiological responses of both species to the combined effect of light and pCO_2_ on elemental composition and photophysiology were assessed.

## Material and Methods

### Culture conditions

Experiments were conducted with the two Antarctic diatom species *Fragilariopsis curta* (isolated during the Polarstern expedition ANT XVI/3, 1999) and *Odontella weissflogii* (isolated during the Polarstern cruise ANT‐XXIX/5, 2013). Two months prior to the start of the experiment, both cultures were grown semi‐continuously at 0°C and 100 μmol photons · m^−2^ · s^−1^ in the laboratory of Rothera station on Adelaide Island, WAP. *Odontella weissflogii* and *F. curta* are commonly found in coastal WAP waters such as Ryder Bay, where they significantly contribute to overall biomass (Garibotti et al. 2003a,b, 2005, Annett et al. 2010). Both species were cultured in sterile‐filtered (0.2 μm) natural unbuffered seawater (salinity: 32.85; silicate: 45 μmol · L^−1^), which was sampled from the Rothera Time Series site 1 (RaTS1) in Ryder Bay. After filtration, this seawater was enriched with nitrate (100 μmol · L^−1^) and phosphate (6.25 μmol · L^−1^) following the Redfield N:P ratio (Redfield 1958) in order to prevent nutrient exhaustion.

Prior to the start of the main experiment, both species were grown outdoors for 14 d under the two dynamic light and two pCO_2_ conditions to preacclimate them. Only during this phase, cultures were diluted with pre‐equilibrated seawater. For the main experiment, *Odontella weissflogii* and *Fragilariopsis curta* were grown in dilute batch cultures outdoors at ~30% (HSR = 451 ± 170 μmol photons · m^−2^ · s^−1^) and ~10% (moderate solar radiation, MSR = 101 ± 51 μmol photons · m^−2^ · s^−1^) of incident solar radiation. In addition to this, the culture medium (without cells) and cultures (medium with cells) were bubbled with either ambient air (325 μatm, current pCO_2_ treatment; using an air‐pump) or premixed‐air of elevated pCO_2_ (800 μatm, future pCO_2_ treatment; Air Liquide Deutschland Ltd., Bremen, Germany). To ensure constant carbonate chemistry over the whole duration of the experiment, the pH (NBS scale) was measured every day in all bubbled culture medium and culture bottles. All triplicates were grown in sterile 2.5 L polycarbonate‐bottles that were kept outdoors in two acrylic glass incubators (115 × 65 × 65 cm) covered with neutral density light filters generating the two distinct daily mean light conditions. The light ranges of the MSR (41–286 μmol photons · m^−2^ · s^−1^) and the HSR (251–1,594 μmol photons · m^−2^ · s^−1^) treatments were comparable to average daily irradiances measured during spring and summer in the upper surface waters around the WAP (Young et al. 2015, 100–700 μmol photons · m^−2^ · s^−1^, Rothera Times Series: 30–1,400 μmol photons · m^−2^ · s^−1^). The experiment was conducted in March 2015 under a natural daily light/dark cycle of 13:11 h. To keep temperatures constant inside the incubators, incubation bottles were cooled by a flow‐through of seawater from the adjacent Ryder Bay (−0.38 ± 0.14°C). In each of the two incubators, incident irradiance and temperature were monitored every 15 min using a PAR (Odyssey Photosynthetic Irradiance Logger, Dataflow Systems PTY Ltd, Christchurch, New Zealand) and a temperature logger (TidbiT, HOBO ware, Onset Computer Corporation, Bourne, MA, USA) over the whole duration of the experiment.

### Seawater carbonate system

The seawater carbonate system was determined based on pH, total alkalinity (TA), silicate, phosphate, temperature, and salinity measurements using the CO2Sys program (Pierrot et al. 2006). To this end, the equilibrium constant of Mehrbach et al. (1973) refitted by Dickson and Millero (1987) was applied. During the experiment, the pH (NBS scale) was measured every day using a calibrated pH‐ion meter (826 pH mobile, Metrohm, Filderstadt, Germany). Calibration (3‐point calibration) was carried out with National Institute of Standards and Technology‐certified buffer systems before use. On the final day of the experiment, TA samples were filtered (Whatman GF/F glass fiber filters, 0.7 mm), poisoned with 0.03% HgCl_2_, and stored at 4°C in 200 mL borosilicate flasks until measurement. Duplicates of TA samples were measured by potentiometric titrations (TW alpha plus; SI Analytics, Weilheim, Germany; Brewer et al. 1986). A certified reference material (provided by Prof. A. Dickson, Scripps, USA; batch no. 111; reproducibility ±13 μmol · kg^−1^) was used to correct for systematic errors. The pCO_2_ treatments were distinct throughout the preacclimation phase and the main experiment as shown in Table 1.

**Table 1:** Partial pressures of CO_2_ (pCO_2_) and dissolved inorganic carbon (DIC) concentrations were calculated from measured total alkalinity (TA), pH, silicate, phosphate, temperature, and salinity using the CO2Sys program (Pierrot et al. 2006). For all bubbled culture medium (without cells) and culture (with cells) bottles, pH (NBS scale) was measured every day over the whole duration of the experiment, whereas TA was measured only at the final day of the experiment. Values represent the means (±SD) of each respective parameter from all samplings over the course of the experiments

Target pCO_2_ (μatm)	pCO_2_ (μatm)		TA (μmol · kg^−1^)		pH (NBS)		DIC (μmol · kg^−1^)	
	Medium	Culture	Medium	Culture	Medium	Culture	Medium	Culture
Current, 390	320 ± 4	325 ± 3	2,443 ± 50	2,464 ± 5	8.117 ± 0.005	8.114 ± 0.004	1,701 ± 3	1,709 ± 4
Future, 800	760 ± 13	830 ± 8	2,447 ± 24	2,464 ± 5	7.773 ± 0.001	7.740 ± 0.003	1,818 ± 24	1,832 ± 5

### Cell density

Cell densities were determined from samples that were fixed with Lugol solution (4% final concentration) and stored at 4°C in the dark. After sedimentation for 24 h in a 10 mL Utermöhl chamber (Hydro‐Bios), for each sample >400 cells were counted using an inverted microscope (Axio Observer, D1; Zeiss, Jena, Germany). At the end of the experiment, cell density ranged between 8,695 and 55,267 cells · mL^−1^ for *F. curta*, and 150 and 830 cells · mL^−1^ for *O. weissflogii*. Cell length and width of each species were measured, and did not change between the different treatments (*Fragilariopsis curta*: 3.4 ± 0.7 and 35.3 ± 4.4 μm, *Odontella weissflogii*: 51.0 ± 5.0 and 2.7 ± 0.4 μm). Cell volume was determined following Hillebrand et al. (1999) and accounted on average 10.9 ± 2.8 μm^3^ for *F. curta* and 42.4 ± 6,4 μm^3^ for *O. weissflogii*.

### Elemental composition

For the determination of particulate organic carbon and nitrogen (POC, PON) contents, all cultures were gently filtered (<20 mmHg) onto precombusted glass fiber filters (15 h, 200°C, GF/F) and stored at −20°C for later analysis. Prior to analysis of POC and PON on an elemental analyzer (EURO EA Elemental Analyzer, Euro Vector, Redavalle, Italy), samples were defrosted (>12 h, 60°C), acidified with 0.1 mol HCl · L^−1^, and dried overnight (>12 h, 60°C). POC and PON contents were corrected for blank measurements and normalized to filtered volume and cell densities in order to derive cellular quotas of POC and PON.

### Pigments

After gentle filtration onto glass fiber filters (<20 mmHg, GF/F), samples were immediately frozen and stored at −80°C until analysis. Pigments were extracted with 90% acetone (v/v) for 24 h at 4°C in the dark. Total pigment concentrations were determined via high‐performance liquid chromatography (LaChromElite^®^ system, VWR, Darmstadt, Germany) using a Spherisorb ODS‐2 column (5 μm particle size; Waters, Milford, MA, USA) and applying a gradient following Wright et al. (1991). Peaks were identified and quantified via cochromatography of pigment standards (DHI Lab Products, Hørsholm, Denmark) and the software EZChrom Elite ver. 3.1.3. Cellular pigments were separated into the categories light‐harvesting pigments (LHP: chlorophyll *a* [Chl *a*], chlorophyll c_2_, fucoxanthin) and light‐protective pigments (LPP: diadinoxanthin and diatoxanthin). Pigment contents were normalized to filtered volume and cell densities to yield cellular quotas.

### Chlorophyll *a* fluorescence

Chl *a* fluorescence was measured with a Fast Repetition Rate fluorometer (FRRf, FastOcean PTX; Chelsea Technologies, West Molesey, UK) and a FastAct Laboratory system (Chelsea Technologies) using single turnover saturation and the settings described in Heiden et al. (2016). Samples were dark‐acclimated for 10 min prior to the measurement. Minimum (F_0_) and maximum Chl *a* fluorescence (F_m_) were based on iterative algorithms for induction (Kolber et al. 1998) and relaxation phase (Oxborough et al. 2012). The dark‐adapted maximum PSII quantum yield (F_v_/F_m_) was calculated as:

(1) $$F_{v}/F_{m}=\left(F_{m}-F_{0}\right)/F_{m}$$

Additional Chl *a* fluorescence measurements were performed at 0°C on every treatment in response to increasing incident irradiances (E; μmol photons · m^−2^ · s^−1^) generating photosynthesis–irradiance curves (PE curves; irradiances ranged between 0 and 927 μmol photons · m^−2^ · s^−1^) using seven steps with an acclimation duration of 5 min per light step and with six subsequent Chl *a* fluorescence measurements. From the fluorescence measurements, the light‐adapted minimum (F’) and maximum (F_m_’) Chl *a* fluorescence were derived to calculate the effective PSII quantum yield under ambient light (Genty et al. 1989).

(2) $$F_{q}^{\prime }/F_{m}^{\prime }=\left(F_{m}^{\prime }-F^{\prime }\right)/F_{m}^{\prime }$$

Absolute electron transport rates (absETR) were calculated from the dark‐adapted functional absorption cross‐section of PSII photochemistry (σ_PSII_) and the incident irradiance (E) according to the following equation (Suggett et al. 2004, 2009, Schreiber et al. 2012):

(3) $$\text{absETR}=\sigma_{\mathrm{PSII}}\times \left(\left(F_{q}^{\prime }/F_{m}^{\prime }\right)/\left(F_{v}/F_{m}\right)\right)\times E$$

Using the SigmaPlot 12.3 software (SysStat Software Inc., San Jose, CA, USA), the irradiance‐dependent absETRs were fitted following Ralph and Gademann (2005). The following light‐use characteristics were determined: maximum light‐use efficiency (α), minimum saturating irradiance (I_K_), and maximum absolute electron transport rate (ETR_m_). From the single turnover measurements of dark‐adapted cells, the dark‐adapted functional absorption cross‐section of PSII photochemistry (σ_PSII_, nm^2^ · PSII^−1^), the dark‐adapted re‐oxidation time of the electron acceptor Q_A_ (τ_QA_, μs), the connectivity factor (*p*, dimensionless) of adjacent PSII light‐harvesting pigment matrices, and the concentration of functional PSII reaction centers ([RCII]; amol per cell) were derived according to Oxborough et al. (2012), using the FastPro8 software (Version 1.0.50; Kevin Oxborough, CTG Ltd., West Molesey, UK).

### Statistics

All data are given as means (*n* = 3) ± SD. Normality of data was tested using the Shapiro–Wilk test. To test for significant effects of pCO_2_, light *t*‐tests (level of significance *P* < 0.05) were performed. In case of non‐normality of data, rank sum tests were conducted (Mann–Whitney). Statistical analyses were performed with SigmaPlot 12.3 (SysStat Software Inc., San Jose, CA, USA). In all tables and figures, significant differences (*P* < 0.05) between treatments were indicated by ^+^ for light effects and * for pCO_2_ effects and are summarized in Table 2.

**Table 2:** Significant differences (level of significance *P* < 0.05) between treatments for all parameters were assessed using *t*‐tests and are indicated by ^+^ for light effects (MSR vs. HSR) and * for pCO_2_ effects (current vs. future)

Parameter	Species	Light effect	Light effect	pCO_2_ effect	pCO_2_ effect
		Within current	Within future	Within MSR	Within HSR
POC	*Fragilariopsis curta*	+		*	*
	*Odontella weissflogii*	+		*	*
PON	*F. curta*	+		*	*
	*O. weissflogii*	+		*	*
C:N	*F. curta*			*	
	*O. weissflogii*				*
LHP	*F. curta*	+			*
	*O. weissflogii*	+			
LPP	*F. curta*		+		*
	*O. weissflogii*	+		*	
LHP:LPP	*F. curta*	+	+		
	*O. weissflogii*	+		*	
F_v_/F_m_	*F. curta*			*	*
	*O. weissflogii*			*	*
ETR_m_	*F. curta*	+			*
	*O. weissflogii*	+		*	
I_K_	*F. curta*	+		*	*
	*O. weissflogii*	+			
α	*F. curta*				
	*O. weissflogii*				
σ_PSII_	*F. curta*		+	*	
	*O. weissflogii*				
*p*	*F. curta*			*	
	*O. weissflogii*			*	*
τ_QA_	*F. curta*				
	*O. weissflogii*				
[RCII]^cell^	*F. curta*				
	*O. weissflogii*				
(Chl *a*+c_2_): [RCII]	*F. curta*				
	*O. weissflogii*				

## Results

### Cellular elemental composition

**Figure 1:**
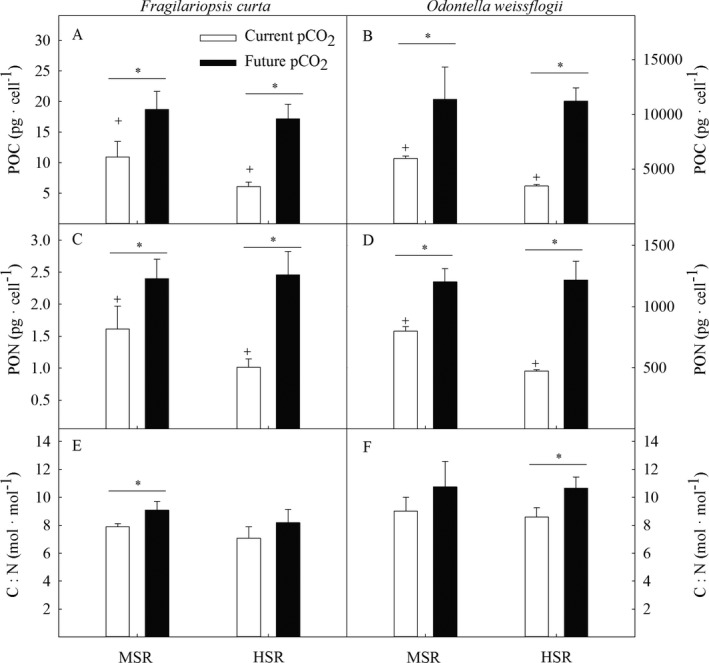
Cellular quotas of particulate organic carbon (POC, pg · cell^−1^; A, B) and particulate organic nitrogen quotas (PON, pg · cell^−1^; C, D), as well as molar ratios of carbon to nitrogen (C:N, mol · mol^−1^; E, F) were determined for *Fragilariopsis curta* and *Odontella weissflogii* acclimated to different light (MSR, moderate solar radiation; HSR, high solar radiation) and pCO_2_ (current = 390 μatm and future = 800 μatm) conditions. Values represent mean ± SD (*n* = 3). Significant differences (*P* < 0.05) between treatments are indicated by ^+^ for light effects and * for pCO_2_ effects.

In both species, cellular quotas of POC and PON, respectively, decreased under current pCO_2_ from MSR to HSR (Fig. 1, Table 2). Within both light treatments of the two species, quotas of POC and PON significantly increased from current to future pCO_2_. Cell size of both species was not affected by the applied treatments (data not shown). The carbon to nitrogen ratio (C:N) was not influenced by the applied light treatments (Fig. 1). Yet, from current to future pCO_2_, C:N ratios increased by 13% in *Fragilariopsis curta* at MSR and by 19% in *Odontella weissflogii* at HSR.

### Cellular pigment contents

In *Fragilariopsis curta*, under current pCO_2_, the concentration of light‐harvesting pigments per cell (LHPs: Chl *a*, chlorophyll c_2_, and fucoxanthin) was significantly reduced from MSR to HSR (Table 3). In this species, future pCO_2_ further led to significantly higher contents of LHP, but only in HSR treatments. In *Odontella weissflogii*, the concentration of LHPs remained constant across light and pCO_2_ treatments. From MSR to HSR, cellular photoprotective pigment contents (LPPs: diadino‐ and diatoxanthin) increased from MSR to HSR in *Fragilariopsis curta* under future pCO_2_ and in *Odontella weissflogii* under current pCO_2_ (Table 3). In *O. weissflogii* grown at MSR, cellular LPP concentrations increased significantly from current to future pCO_2_. From current to future pCO_2_, cellular LPP concentrations remained unaffected under MSR in *F. curta*, but increased at HSR. The ratio of cellular light‐harvesting to photoprotective pigment concentrations (LHP:LPP) generally decreased from MSR to HSR in both species irrespective of the pCO_2_ (Table 3). Only in the future pCO_2_ treatments of *O. weissflogii*, the LHP:LPP ratio did not change from MSR to HSR. Furthermore, in *O. weissflogii* when grown under MSR, the LHP:LPP ratio declined by 49% from current to future pCO_2_.

**Table 3:** Cellular concentrations (in fg · cell^−1^) of light‐harvesting pigments (LHP: sum of chlorophyll *a*, chlorophyll c_2_, and fucoxanthin) and light‐protective pigments (LPP: sum of diadinoxanthin and diatoxanthin), as well as the ratio of cellular contents of light‐harvesting to light‐protective pigments (LHP:LPP) of *Fragilariopsis* and *Odontella* acclimated to different solar radiation (MSR, moderate solar radiation; HSR, high solar radiation) and pCO_2_ (current = 390 μatm and future = 800 μatm) conditions. Values represent mean ± SD (*n* = 3). Significant differences (*P* < 0.05) between treatments are indicated by ^+^ for light effects and * for pCO_2_ effects

pCO_2_	LHP		LPP		LHP:LPP	
	Current	Future	Current	Future	Current	Future
*Fragilariopsis curta*						
MSR	144 ± 27^+^	133 ± 5	4 ± 1	4 ± 1^+^	35 ± 4^+^	34 ± 6^+^
HSR	99 ± 5*^,+^	152 ± 12*	5 ± 0*	8 ± 1*^,+^	19 ± 2^+^	18 ± 1^+^
*Odontella weissflogii*						
MSR	50,118 ± 4,292^+^	44,171 ± 7,538	1,264 ± 33*^,+^	1,979 ± 22*	43 ± 11*^,+^	22 ± 1*
HSR	40,123 ± 873^+^	39,900 ± 4,186	2,101 ± 38^+^	2,096 ± 55	19 ± 1^+^	19 ± 2

### Chlorophyll *a* fluorescence

**Figure 2:**
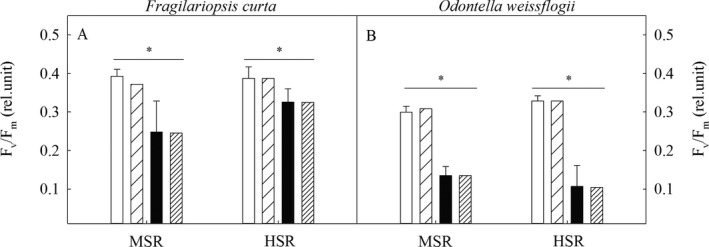
Dark‐adapted maximum photosystem II quantum yield (F_v_/F_m_, dimensionless; A, B) were determined for *Fragilariopsis curta* and *Odontella weissflogii* acclimated to different light (MSR, moderate solar radiation; HSR, high solar radiation) and pCO_2_ (current= 390 μatm and future= 800 μatm) conditions. Modeled F_v_/F_m_ values are presented (dashed bars, A and B) for comparison with the measured F_v_/F_m_ (filled bars). Values represent mean ± SD (*n* = 3). Significant differences (*P* < 0.05) between treatments are indicated by ^+^ for light effects and * for pCO_2_ effects.

In both species, the dark‐adapted maximum PSII quantum yield (F_v_/F_m_) was not affected by the applied light treatments (Fig. 2, A and B). However, in the two species from current to future pCO_2_, F_v_/F_m_ significantly decreased under both light treatments. In *Fragilariopsis curta* and *Odontella weissflogii*, the maximum electron transport rate (ETR_m_) and minimum saturating irradiance (I_K_), derived from PE curve fits, increased from MSR to HSR under current pCO_2_ (Table 4). Under future pCO_2_, no light effects in ETR_m_ and I_K_ were found in both species. From current to future pCO_2_, in *F. curta*, I_K_ increased by 61% at MSR while at HSR next to I_K_ also ETR_m_ decreased (45% and 39%, respectively). In *O. weissflogii*, ETR_m_ increased from current to future pCO_2_ at MSR, but not at HSR. Even though *F. curta* and *O. weissflogii* showed different light‐ and CO_2_‐dependent responses in ETR_m_ and I_K_ the ratio thereof, however, remained unchanged, thus resulting in an unaltered light‐use efficiency (α; Table 4). In *F. curta*, the dark‐adapted functional absorption cross‐section of PSII photochemistry (σ_PSII_), a measure for the total area of light harvesting, was lower at MSR when combined with future pCO_2_ than in all other treatment combinations (Table 4). In *O. weissflogii*, σ_PSII_ was neither influenced by pCO_2_ nor by light. The connectivity of adjacent photosystems (*p*) decreased from current to future pCO_2_ in both light treatments of *O. weissflogii*, but only in the MSR treatment of *F. curta* (Table 4). Within the current and future pCO_2_ treatments, increasing light did not affect *p*. In both species, the dark‐adapted re‐oxidation time of the electron acceptor Q_A_ (τ_QA_) remained constant across all treatments (Table 4). In *Fragilariopsis curta* and *Odontella weissflogii*, neither solar radiation nor pCO_2_ did affect the number of functional PSII reaction centers ([RCII]^cell^; Table 4). The amount of all chlorophylls (Chl *a* and Chl c_2_) normalized to functional PSII reaction centers ((Chl *a*+c2):[RCII]; Table 4) remained unchanged for all treatments in both species.

**Table 4:** Photosynthesis‐related parameters of *Fragilariopsis curta* and *Odontella weissflogii* acclimated to different solar radiation (MSR, moderate solar radiation; HSR, high solar radiation) and pCO_2_ (current = 390 μatm and future = 800 μatm) conditions were derived from irradiance‐dependent absETRs curve fits following Ralph and Gademann (2005). Given are maximum electron transport rate (ETR_m_, e^−^ · PSII^−1^ · s^−1^), minimum saturating irradiance (I_K_, μmol photons · m^−2^ · s^−1^), and light‐use efficiency (α, rel. unit), the dark‐adapted functional absorption cross‐section of PSII (σ_PSII_, nm^2^ per quanta), the connectivity factor (*p*, dimensionless) of adjacent PSII light‐harvesting pigment matrices, the dark‐adapted re‐oxidation of the electron acceptor Q_A_ (τ_QA_, μs), the cellular concentration of functional PSII reaction centers ([RCII]^cell^, amol per cell), and the number of chlorophylls *a* and c_2_ per functional PSII reaction centers ((Chl *a*+c_2_):[RCII], mol · mol^−1^). Values represent mean ± SD (*n* = 3). Significant differences (*P* < 0.05) between treatments are indicated by ^+^ for light effects and * for pCO_2_ effects

pCO_2_	ETR_m_		I_K_		α		σ_PSII_	
	Current	Future	Current	Future	Current	Future	Current	Future
*F. curta*								
MSR	175 ± 39^+^	211 ± 73	44 ± 8*^,+^	71 ± 7*	4.1 ± 1.1	3.0 ± 1.0	8.1 ± 0.7*	6.5 ± 0.3*^,+^
HSR	391 ± 68*^,+^	237 ± 45*	134 ± 25*^,+^	74 ± 10*	3.0 ± 0.6	3.2 ± 0.4	8.6 ± 0.6	8.2 ± 0.3^+^
*O. weissflogii*								
MSR	391 ± 77*^,+^	657 ± 143*	166 ± 32^+^	245 ± 63	2.4 ± 0.2	2.7 ± 0.2	4.0 ± 0.5	4.0 ± 0.3
HSR	764 ± 147^+^	438 ± 420	297 ± 63^+^	175 ± 83	2.6 ± 0.3	2.0 ± 1.2	4.9 ± 1.1	4.2 ± 0.5

	*p*		τ_QA_		[RCII]^cell^		(Chl *a*+c_2_):[RCII]	
	Current	Future	Current	Future	Current	Future	Current	Future
*F. curta*								
MSR	0.31 ± 0.05*	0.17 ± 0.04*	502 ± 25	486 ± 88	0.06 ± 0.02	0.09 ± 0.03	1,699 ± 142	1,078 ± 392
HSR	0.32 ± 0.03	0.26 ± 0.05	474 ± 12	500 ± 39	0.05 ± 0.01	0.08 ± 0.01	1,506 ± 443	1,291 ± 299
*O. weissflogii*								
MSR	0.27 ± 0.03*	0.11 ± 0.01*	593 ± 20	503 ± 63	23.07 ± 2.20	40.18 ± 2.74	1,448 ± 223	766 ± 316
HSR	0.29 ± 0.01*	0.11 ± 0.07*	599 ± 19	736 ± 142	20.16 ± 0.93	42.23 ± 34.02	1,311 ± 93	1,044 ± 955

## Discussion

In this study, the two bloom‐forming Antarctic diatom species *Fragilariopsis curta* and *Odontella weissflogii* were grown under moderate and high incident solar radiation (MSR = 101 ± 50 μmol photons · m^−2^ · s^−1^ and HSR = 451 ± 170 μmol photons · m^−2^ · s^−1^, respectively) combined with either current or future pCO_2_ (325 and 800 μatm, respectively). The effects of increasing pCO_2_ on cellular POC quotas and photophysiology were found to be stronger than those exerted by increasing natural solar radiation. In the following, we will first elucidate whether the previously observed physiological responses to increasing constant light in *F. curta* and *O. weissflogii* (Heiden et al. 2016) are representative also for increasing natural solar radiation and will assess their detailed effects on diatom physiology. As found here that pCO_2_ effects were similar irrespective of the applied solar radiation regime, they will therefore be jointly discussed. In order to investigate whether dynamic light modulates the responses of Antarctic diatoms to future pCO_2_, results from this study are further compared to the previously observed OA‐dependent changes in *F. curta* and *O. weissflogii* under constant light (Heiden et al. 2016).

### Increasing daily integrated irradiance of solar radiation reduced cellular POC contents

Variable light regimes create a continuous alternation between unfavorable (over‐ and under‐saturation) and favorable conditions for photosynthesis. Therefore, a phytoplankton cell continuously has to adjust to the changing light conditions, which can reduce the energy transfer efficiency from photosynthesis to carbon fixation (Wagner et al. 2006, Shatwell et al. 2012, Su et al. 2012, Hoppe et al. 2015). In this study, under both light regimes C:N ratios of the two investigated species ranged between 7 and 9 mol · mol^−1^ at current pCO_2_ (Fig. 1), thus being above the Redfield ratio of 6.6 mol · mol^−1^ typically reported for marine phytoplankton (Redfield 1958). The here measured C:N ratios of the tested species were much higher when compared to the values previously measured under the constant irradiances of 200 and 500 μmol photons · m^−2^ · s^−1^ (4.5–6.0 mol · mol^−1^ for *Fragilariopsis curta* and 4.9–6.5 mol · mol^−1^ for *Odontella weissflogii*; Heiden et al. 2016). These elevated C:N ratios can be attributed to much higher cellular POC quotas under the here applied solar radiation regimes (Fig. 1) compared to the rather low POC quotas of 4.8 and 4.3 pg · cell^−1^ in *F. curta* and 3,205 and 1,889 pg · cell^−1^ in *O. weissflogii* estimated previously under the constant irradiances of 200 and 500 μmol photons · m^−2^ · s^−1^ (J.P. Heiden, unpubl. data). Hence, exposure to solar radiation regimes led to enhanced cellular carbon build‐up in our two tested diatom species compared to their exposure to similar constant light conditions.

In this study, the cellular quotas of POC and PON, however, decreased from MSR to HSR in both species (POC and PON ~40%; Fig. 1), indicating a negative effect of increasing daily integrated irradiances under the same solar radiation regime. Reduced POC contents under increasing dynamic light from 65 to 125 or 250 μmol photons · m^−2^ · s^−1^ were previously found for the Antarctic diatom *Fragilariopsis cylindrus* (Mills et al. 2010). In line with this, under constant irradiances cellular POC quotas (J.P. Heiden, unpub. data) and POC production rates (Heiden et al. 2016) also declined from moderate to high light (200–500 μmol photons · m^−2^ · s^−1^) in *F. curta* and *Odontella weissflogii*. This was likely due to light stress under these high irradiances. Hence, as previously observed, the amount of variability in light, including constant, sine and more dynamic light conditions, impact how much of the absorbed photons are converted into biomass with substantial differences between light regimes (Wagner et al. 2006, Arrigo et al. 2010, Hoppe et al. 2015, Lepetit et al. 2016, Lin et al. 2016). The lowered POC contents from MSR to HSR in the two tested species were further accompanied by increased maximum electron transport rates under current pCO_2_ (ETR_m_; Table 4), indicating no light saturation of absETRs. Similarly, decreased or unaffected POC production rates together with increased ETR_m_ were previously found in *F. curta* and *O. weissflogii* under moderate compared to low constant light (Heiden et al. 2016; 20 and 200 μmol photons · m^−2^ · s^−1^) and in *Chaetoceros debilis* under dynamic compared to constant irradiances (Hoppe et al. 2015, 90 μmol photons · m^−2^ · s^−1^). In *Chaetoceros brevis*, cellular POC quotas remained unaffected while short‐term photosynthetic oxygen evolution rates increased with increasing irradiance in a dynamic light regime (Boelen et al. 2011, 76 and 200 μmol photons · m^−2^ · s^−1^). Only for *F. cylindrus*, no effects of increasing daily mean irradiances on ETR_m_ and cellular POC quotas were observed under dynamic light (Mills et al. 2010, 125 and 250 μmol photons · m^−2^ · s^−1^). Hence, in response to increasing daily irradiances of either constant or dynamic light photosynthetic electron transport rates increased in our two tested species while POC concentrations remained unaffected or even decreased under these conditions. This could be due to a saturation of the Calvin–Benson cycle, which is generally considered to be the rate‐limiting step of photosynthesis under excessive light conditions. Increasing ETR_m_ at unaffected POC production would thus create the demand for operation of alternative electron pathways such as cyclic electron transport around PSI to dissipate excess electrons (Behrenfeld and Milligan 2012). Very active cyclic electron flow around PSI was previously reported for the Antarctic diatom *F. cylindrus* and an Antarctic diatom‐dominated natural phytoplankton community (Goldman et al. 2015). The cyclic electron flow was supposedly most intense during midday and thus during periods of high light. In the diatom *Phaeodactylum tricornutum*, increased cyclic electron transport around PSI (photosynthetic electrons not used in biomass formation) was also observed with increasing daily mean irradiances (Wagner et al. 2006). This would raise the transthylakoid pH gradient and lead to a higher production of ATP at the expense of NADPH, thus potentially explaining the observed lowered POC quotas in this study (Falk and Palmqvist 1992).

The dark‐adapted maximum PSII quantum yield (F_v_/F_m_) is considered as an indicator for physiological stress. In a previous study, no effect of increasing dynamic light on F_v_/F_m_ was reported for the Antarctic diatom *Chaetoceros brevis* (Boelen et al. 2011). In comparison, F_v_/F_m_ decreased from moderate to high daily integrated intensities of dynamic light in the Antarctic diatom *Fragilariopsis cylindrus*, indicating light stress (Kropuenske et al. 2009, Mills et al. 2010). In our two species here, F_v_/F_m_ was not affected from MSR to HSR (*F. curta*: 0.39 and 0.39; *Odontella weissflogii*: 0.30 and 0.33; Fig. 2). Interestingly, when grown under constant light, the small *F. curta* exhibited a higher susceptibility to stress already at lower constant irradiances than the much larger *O. weissflogii* (Heiden et al. 2016). We compared the here measured F_v_/F_m_ values under dynamic light to those estimated previously for the same species under moderate and high constant light (*F. curta*: 0.36 and 0.36; *O. weissflogii*: 0.35 and 0.45; Heiden et al. 2016). This comparison shows that the F_v_/F_m_ values of *O. weissflogii* in this study were overall lower than under constant light, indicating that the latter is more prone for light stress resulting from dynamic than from constant light exposure. This was surprisingly not the case for the small *F. curta* when grown under dynamic compared to constant light, indicating a higher tolerance to cope with fluctuating light regimes than *O. weissflogii*. A higher susceptibility to increasing irradiances in small cells was reported previously (Raven 1984, 1991, Karentz et al. 1991, Garcia‐Pichel 1994, Raven and Kübler 2002, Key et al. 2010).

A shift from light harvesting to photoprotection (LHP:LPP; Table 3) occurred from MSR to HSR in both species, caused by reduced cellular contents of LHPs a common response in diatoms to increasing constant (Heiden et al. 2016, Trimborn et al. 2017) or dynamic light (Kropuenske et al. 2009, Boelen et al. 2011). In line with our results (Table 3), the potential for dissipation of excess light energy (cellular LPP contents) remained unaffected with increasing dynamic light in *Fragilariopsis cylindrus* and *Chaetoceros brevis* (Kropuenske et al. 2009, Boelen et al. 2011). Furthermore, in these two Antarctic diatoms cellular LPP contents were found to be higher under dynamic compared to constant light of the same daily mean irradiance (Kropuenske et al. 2009, Mills et al. 2010, Boelen et al. 2011). Congruently, LPP contents estimated for *Fragilariopsis curta* at MSR and HSR in this study (4 ± 1 and 5 ± 0 fg · cell^−1^; Table 3) were significantly higher when compared to values previously estimated under constant moderate and high light (Heiden et al. 2016, 2.4 ± 0.6 and 3.5 ± 0.3 fg · cell^−1^). We therefore suggest that the fluctuation of light rather than the mean daily irradiance controlled the photoprotective pigment composition in *F. curta*. Interestingly, in *Odontella weissflogii*, LPP concentrations were generally higher under constant (Heiden et al. 2016, 5,300 ± 500 and 2,900 ± 200 fg · cell^−1^ at 200 and 500 μmol photons · m^−2^ · s^−1^, respectively) than under dynamic light (MSR = 1,264 ± 33 fg · cell^−1^ and HSR = 2,101 ± 38 fg · cell;^−1^ Table 3) of comparable daily mean irradiances. In line with this, despite apparent light stress under high compared to moderate constant irradiances, cellular LPP contents of *O. weissflogii* were reduced (Heiden et al. 2016) indicating different ways to counteract light stress in this species when compared to *F. curta*.

Overall, the effects of increasing irradiances in dynamic light regimes on diatom physiology are similar to strategies applied when grown under increasing constant light regimes. The saturation of the Calvin–Benson cycle was also the rate‐limiting step of photosynthesis under higher growth irradiances, thus increasing the demand for alternative ways to dissipate excess electrons. Furthermore, dynamic compared to constant light of comparable daily mean irradiances (dynamic: 101 and 451 μmol photons · m^−2^ · s^−1^; constant: 200 and 500 μmol photons · m^−2^ · s^−1^) was more stressful for the tested Antarctic diatoms. In the dynamic light treatments, light stress occurred already at lower mean light intensities. Species‐specific strategies to counteract this enhanced light stress under dynamic irradiances, however, existed as observed by the different regulation of LPP contents by *Fragilariopsis curta* and *Odontella weissflogii*.

### Future pCO_2_ stimulated carbon build‐up, but decreased photosystem efficiency

In line with observations from tropical phytoplankton assemblages (Biswas et al. 2017), this study found a positive effect of future pCO_2_ on cellular POC and PON quotas in both species and under both solar radiation regimes (Fig. 1). The latter response was unexpected, considering that future pCO_2_ did not affect POC quotas in several Antarctic diatoms when they were grown at constant light (Boelen et al. 2011, Hoppe et al. 2015, Heiden et al. 2016, Trimborn et al. 2017). Also, under dynamic light, POC quotas in *Chaetoceros brevis* remained unaffected (Boelen et al. 2011), but declined in *C. debilis* (Hoppe et al. 2015). Hence, the few studies conducted so far reported no or negative OA effects on cellular POC quotas of Antarctic diatoms grown under dynamic irradiances. Therefore, the here OA‐dependent stimulation in POC accumulation was surprising. This positive effect was, however, accompanied by an OA‐dependent decline in F_v_/F_m_ in both species irrespective of the applied solar radiation regimes (Fig. 2). Hence, future pCO_2_ was stressful for the two investigated species. In line with this, in *F. curta* under HSR and in *O. weissflogii* under MSR, cellular LPP contents were also enhanced (Table 3), indicating higher demands for the dissipation of excess excitation energy under elevated pCO_2_.

Interestingly, also the re‐oxidation time of the electron acceptor Q_A_ (τ_QA_), remained unaltered irrespective of the applied pCO_2_ and light treatments (Table 4). τ_QA_ indicates how fast the absorbed energy can be transferred from PSII to Q_A_ and thus into downstream processes of photosynthesis. Therefore, we suggest that the here observed decrease in F_v_/F_m_ under future pCO_2_ was not caused by alterations in downstream processes of photosynthesis. In fact, from current to future pCO_2_, the connectivity between PSIIs (*p*, Table 4) significantly declined in both species and solar radiation regimes (not significant for *Fragilariopsis curta* at HSR). The lower capacity to distribute excitons among PSIIs makes the PSII reaction centers more prone for over‐excitation (Blankenship 2002). As the total area of light harvesting σ_PSII_ also remained unaffected by future pCO_2_ (Table 4), this further implied that a greater proportion of the absorbed energy was lost before an exciton decay yielded reduction in Q_A_ under these conditions.

**Figure 3:**
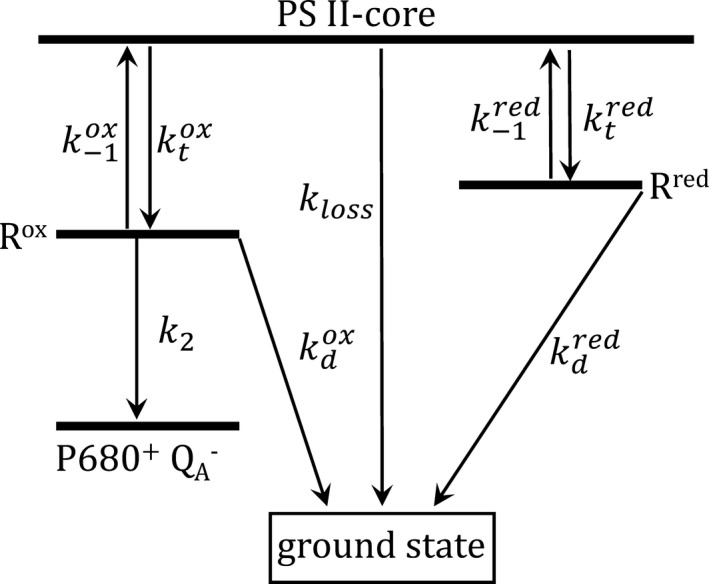
: Exciton‐radical‐pair equilibrium model for the PSII core in the open (Q_A_) and closed (Q_A_ ^‐^) state. *k*_loss_ (Table 5) represents non‐photochemical losses from the antenna. The following rate constants are suggested for modeling of PSII (Trissl and Lavergne 1995, Kroon and Thoms 2006): $k_{t}^{\mathrm{ox}}$ = 540/*N*_II_ ns^−1^, exciton trapping by an open reaction center, depends on the antenna size *N*_II_ (Table 5); *k*_2_ = 2.3 ns^−1^, reduction of Q_A_; $k_{-1}^{\mathrm{ox}}$ = 0.3 ns^−1^, charge recombination in PSII units with open centers; $k_{d}^{\mathrm{ox}}$ = 0.001 ns^−1^, non‐radiative losses from the radical pair (R^ox^) in an open center; $k_{t}^{\mathrm{red}}$ = 84.6/*N*_II_ ns^−1^, exciton trapping by a closed center, depends on the antenna size *N*_II_ (Table 5); $k_{-1}^{\mathrm{red}}$ = 0.34 ns^−1^, charge recombination in PSII units with closed centers; $k_{d}^{\mathrm{red}}$ = 0.99 ns^−1^, non‐radiative losses from the radical pair (R^red^) in a closed center.

To better understand the potential causes for the here observed CO_2_‐dependent decline in F_v_/F_m_ (Fig. 2), we applied a biooptical models to assess primary processes in PSII. This also allows verifying the postulated losses and decay of excitation energy in the antenna of PSIIs. Using the exciton‐radical pair equilibrium model of Lavergne and Trissl (1995) for the description of the overall energy conversion in PSII (Fig. 3), the F_v_/F_m_ was calculated as:

(4a) $$\frac{F_{v}}{F_{m}}=\frac{k_{\mathrm{open}}-k_{\mathrm{closed}}}{k_{\mathrm{open}}+k_{\mathrm{loss}}}$$

(4b) $$k_{\mathrm{open}}=\frac{k_{t}^{\mathrm{ox}}\left(k_{d}^{\mathrm{ox}}+k_{2}\right)}{k_{-1}^{\mathrm{ox}}+k_{d}^{\mathrm{ox}}+k_{2}}$$

(4c) $$k_{\mathrm{closed}}=\frac{k_{t}^{\mathrm{red}}\times k_{d}^{\mathrm{red}}}{k_{-1}^{\mathrm{red}}+k_{d}^{\mathrm{red}}}$$

where *k*_open_ and *k*_closed_ represent the effective overall rate constants. The latter combine the molecular rate constants of deactivation paths of absorbed excitons at open and closed PSII reaction centers, respectively. *k*_loss_ describes the effective loss of excitation energy from the antenna as a whole, including the radiative pathway and energy dissipation by LPPs (DD and DT). The here used values for the exciton‐radical pair model are summarized in the caption of Figure 3. Please note that the rate constants for the trapping of the exciton by the reaction centers depend on the antenna size. This was estimated from the fraction of total light absorbed by PSII (50%; Suggett et al. 2004), which was found to be similar for low and high light growth conditions in diatoms (Suggett et al. 2004). We calculated antenna size from the measured number of Chl *a* and c_2_ per RCII as 0.5 × (Chl *a*+c_2_) × [RCII]^−1^ (*F*_PSII_; Table 5). Furthermore, we assumed that RCII core processes (Fig. 3) remained unaffected by solar radiation and pCO_2_. The measured and calculated (eq. (4a)) F_v_/F_m_ values were very similar in all treatments (Fig. 2). The model found no light effect on *k*_loss_ for both species under current and future pCO_2_ (Table 5), which is in line with our measured F_v_/F_m_ under these conditions (Fig. 2). The model calculations, however, indicated a significant OA‐dependent increase in *k*_loss_, this effect was less pronounced in *Fragilariopsis curta* compared to *Odontella weissflogii* (Table 5). In response to future pCO_2_, an up to 3‐fold increase in *k*_loss_ in *F. curta* can explain the reduction in F_v_/F_m_ under both solar radiation treatments. A similar increase in excitation loss was previously found in two diatoms, but triggered by high light exposure (Miloslavina et al. 2009) while CO_2_‐dependent effects were not investigated. In *O. weissflogii*, we observed a comparably much stronger reduction in the measured F_v_/F_m_ than in *F. curta*. In terms of the model, this was a 9‐fold increase in *k*_loss_ compared to *k* ^0^_loss_ under future pCO_2_ (Table 5). A similarly strong increase in the deactivation rate (on average by a factor of 9) was caused by aggregates of plant light‐harvesting complexes (Lambrev et al. 2011). Therefore, we suggest similar structural rearrangements of the antenna complexes in *O. weissflogii*. The observed CO_2_‐dependent decline in F_v_/F_m_, however, does not explain why under these conditions the cellular POC contents increased. As the number of functional reaction centers ([RCII]^cell^, Table 4) increased with increasing pCO_2_, although this effect was not statistically significant, an increase in the sheer number of [RCII]^cell^ could have compensated for the reduced efficiency of the individual antenna complexes, thereby not only maintaining, but also facilitating enhanced rates of POC build‐up. Thus, future pCO_2_ enhanced photosensitivity of the two tested diatoms, increasing therefore the demand for dissipation of excitation energy and reducing their photochemical yield.

**Table 5:** The number of Chl *a* and Chl c_2_ molecules in PSII (*N*_II_ = Chl *a*+c_2_) were derived from the measured (Chl *a*+c_2_):[RCII] (Table 4) and an estimated proportion of total quanta absorbed by only PSII (*F*_PSII_ in %; Suggett et al. 2004). The non‐photochemical deactivation rate *k*_loss_ (in ns^−1^) of the PSI antenna is compared with the rate = 0.75 ns^−1^, which was measured for diatoms under dark‐adapted conditions (Miloslavina et al. 2009)

pCO_2_	*F*_PSII_		*N*_II_ = Chl *a*+c_2_		$k_{\mathrm{loss}}/k_{\mathrm{loss}}^{0}$	
	Current	Future	Current	Future	Current	Future
*Fragilariopsis curta*						
MSR	50		849	539	1.00	3.00
HSR	50		753	645	1.05	1.65
*Odontella weissflogii*						
MSR	50		724	383	1.60	9.00
HSR	50		655	522	1.60	9.00

## Conclusions

While performance of laboratory experiments under constant light regimes are valuable to decipher the underlying physiological processes at play, future studies should consider also to conduct their experiments under dynamic light or natural solar radiation to obtain more realistic results. This study could show that OA‐dependent stimulation of carbon build‐up and photosensitivity occurs at lower light intensities under dynamic compared to constant (Heiden et al. 2016) light scenarios. The exposure of the two diatom species *Fragilariopsis curta* and *Odontella weissflogii* to increasing solar radiation and pCO_2_ concentrations (325 and 830 μatm) revealed further that both species displayed strong photophysiological efficiency to counteract HSR under high pCO_2_. Irrespective of the solar radiation regime, future pCO_2_ was found to stimulate POC accumulation, suggesting a higher potential for CO_2_ sequestration by the two bloom‐forming Antarctic coastal diatoms. This can have important implications for primary productivity of diatom‐dominated communities in the future Southern Ocean. However, a shoaling of the upper mixed layer by climate warming and concomitant higher daily irradiances are likely to enhance light stress in diatoms. This is due to enhanced demands for dissipation of excitation energy under future pCO_2_ as seen in both of the here investigated diatom species. Such stress responses could be even stronger under a lowered nutrient input scenario in coastal Antarctic waters. Hence, high light stress may potentially decrease their competitiveness toward other less susceptible phytoplankton groups such as the haptophyte *Phaeocystis antarctica*, which was found to be less sensitive to elevated pCO_2_ when combined to high irradiances (Trimborn et al. 2017). In order to assess the impacts from the here observed OA‐dependent changes in physiology on diatom's species competitiveness within natural phytoplankton assemblages, it would be necessary to investigate the combined effects of future pCO_2_ and solar radiation on phytoplankton dynamics in the field.

We thank Britta Meier‐Schlosser and Tina Brenneis for assistance in the laboratory. We further thank the staff and scientists of the British Antarctic Survey, the Royal Netherlands Institute for Sea Research, the Netherlands Organization for Scientific Research, and all staff and scientists at Rothera station for their support to make this work possible. JPH and ST were funded by the Helmholtz association (HGF Young Investigators Group EcoTrace, VH‐NG‐901).
